# A Pumpless and Tubeless Microfluidic Device Enables Extended In Vitro Development of *Cryptosporidium parvum*

**DOI:** 10.1093/ofid/ofae625

**Published:** 2024-10-16

**Authors:** Samantha Gunasekera, Benjamin Thierry, Edward Cheah, Brendon King, Paul Monis, Jillian M Carr, Abha Chopra, Mark Watson, Mark O’Dea, Una Ryan

**Affiliations:** Harry Butler Institute, College of Environmental and Life Sciences, Murdoch University, Murdoch, Western Australia, Australia; Future Industries Institute, University of South Australia, Adelaide, South Australia, Australia; Future Industries Institute, University of South Australia, Adelaide, South Australia, Australia; South Australian Water Corporation, Adelaide, South Australia, Australia; South Australian Water Corporation, Adelaide, South Australia, Australia; College of Medicine and Public Health, Flinders University, Flinders Health and Medical Research Institute, Bedford Park, South Australia, Australia; Immunology and Infectious Diseases, Murdoch University, Murdoch, Western Australia, Australia; Immunology and Infectious Diseases, Murdoch University, Murdoch, Western Australia, Australia; Harry Butler Institute, College of Environmental and Life Sciences, Murdoch University, Murdoch, Western Australia, Australia; Harry Butler Institute, College of Environmental and Life Sciences, Murdoch University, Murdoch, Western Australia, Australia

**Keywords:** *Cryptosporidium*, fluid shear stress, gut-on-chip, HCT-8 cells, in vitro

## Abstract

**Background:**

The enteric parasite *Cryptosporidium* remains a treatment challenge for drinking water utilities globally due to its resistance to chlorine disinfection. However, the lack of an in vitro culture system for *Cryptosporidium* that is both cost-effective and reliable remains a key bottleneck in *Cryptosporidium* research.

**Methods:**

Here we report that the microfluidic culture of human ileocecal colorectal adenocarcinoma (HCT-8) cells under fluid shear stress enables the extended development of *Cryptosporidium parvum*. Specifically, the growth of *C. parvum* in a user-friendly pumpless microfluidic device was assessed using immunofluorescence assays, scanning electron microscopy, and quantitative polymerase chain reaction, which revealed that development continued for 10 days in total.

**Results:**

Oocysts produced within the microfluidic device were infective to fresh HCT-8 monolayers; however, these oocysts were only present at low levels.

**Conclusions:**

We anticipate that such microfluidic approaches will facilitate a wide range of in vitro studies on *Cryptosporidium* and may have the potential to be further developed as a routine infectivity assessment tool for the water industry.

## Teaser

This study reports the extended in vitro development of Cryptosporidium parvum using a microfluidic gut-on-chip. This in vitro co-culture system benefits from the enhanced physiological relevance of bioengineered cell culture systems while remaining user-friendly and inexpensive.

*Cryptosporidium* is an enteric waterborne pathogen and the leading cause of moderate-to-severe diarrhea in young children in Sub-Saharan Africa and South Asia [1, 2]. *Cryptosporidium* has been attributed to ∼12.9 million disability-adjusted life-years (DALYs) lost globally in children <5 years of age [3]. There is currently no vaccine and very limited treatment options available for cryptosporidiosis [4]. Despite the significant global disease burden of cryptosporidiosis, affordable and robust in vitro culture systems for *Cryptosporidium* that support complete development remain inaccessible to most laboratories, which is recognized as a critical bottleneck in the development of better therapeutic interventions [5, 6].

Due to the small size of *Cryptosporidium* oocysts (∼5 µm), high shedding rates, low infectious dose, environmental persistence, and resistance to chlorination [7], *Cryptosporidium* was responsible for 76.5% of waterborne outbreaks caused by protozoan parasites globally between 2017 and 2020 [8]. Of the >45 *Cryptosporidium* species described, *C. hominis* and *C. parvum* are the 2 main species infecting humans and are responsible for almost all waterborne outbreaks attributed to this genus [8–11]. Accurate assessment of the risk to consumers from *Cryptosporidium* oocysts in drinking water and selection of appropriate levels of water treatment require quantification of the infectious fraction of oocysts. Relying solely on total oocyst counts or nucleic acid–based detection and identification methods may significantly overestimate the risk [12]. The importance of accurate risk assessment of drinking water sources is increasing as many countries are moving to adopt a tolerable risk of 10^−6^ DALYs per person per year as the health target for drinking water quality [13, 14]. Cell culture methods for *Cryptosporidium* infectivity determination using the human ileocecal colorectal adenocarcinoma (HCT-8) cell line are the most reliable and widely used [15, 16] and have confirmed that the proportion of infectious oocysts in source water catchments and wastewater treatment plants for drinking water supply can be highly variable [17, 18].

While comparative studies using a wide range of cell lines have indicated that the HCT-8 cell line supports superior *Cryptosporidium* development [19], oocyst formation occurs at negligible levels using this system, and development will typically cease after 72 hours post-infection [20]. Pioneering work utilizing stem cell–derived culturing systems has consistently demonstrated that *Cryptosporidium* fertilization and subsequent oocyst formation can occur under these conditions [21–25], but these approaches remain inaccessible to many labs due to high reagent costs and reliance on primary cells. Bioengineered intestinal models utilizing cell lines have also demonstrated complete *C. parvum* development [26–28]. While these studies have been influential for subsequent in vitro culture systems for *Cryptosporidium* reported in the literature, they are unsuitable for routine *Cryptosporidium* culture due to the high upfront costs for implementation, complexity of the culturing systems, and inability to scale these systems for diverse applications.

The present study proposes the use of a user-friendly gut-on-chip to extend the current applications of the HCT-8 cell line for the study of *Cryptosporidium* biology while maintaining ease of use and low costs. The control of fluid flow, and consequent application of fluid shear stress to transformed cells cultured within microfluidic devices, has been frequently documented to change the phenotype of these cells to reflect more physiologically relevant structure and function [29–32]. To foster implementation in settings with limited microfluidic experience and facilities, a previously reported pumpless and tubeless microfluidic device was used. In this device, a hydrophilic thread is used to drive the fluid flow, enabling the application of a constant fluid shear stress to cell monolayers as previously validated using the human colorectal adenocarcinoma (Caco-2) cell line [33]. The data presented herein demonstrate that *Cryptosporidium* culture in HCT-8 cells using this technology is cost-effective and reliable, with the potential to be widely used for *Cryptosporidium* infectivity determination.

## METHODS

### Fabricating Microfluidic Devices

**Figure 1.**
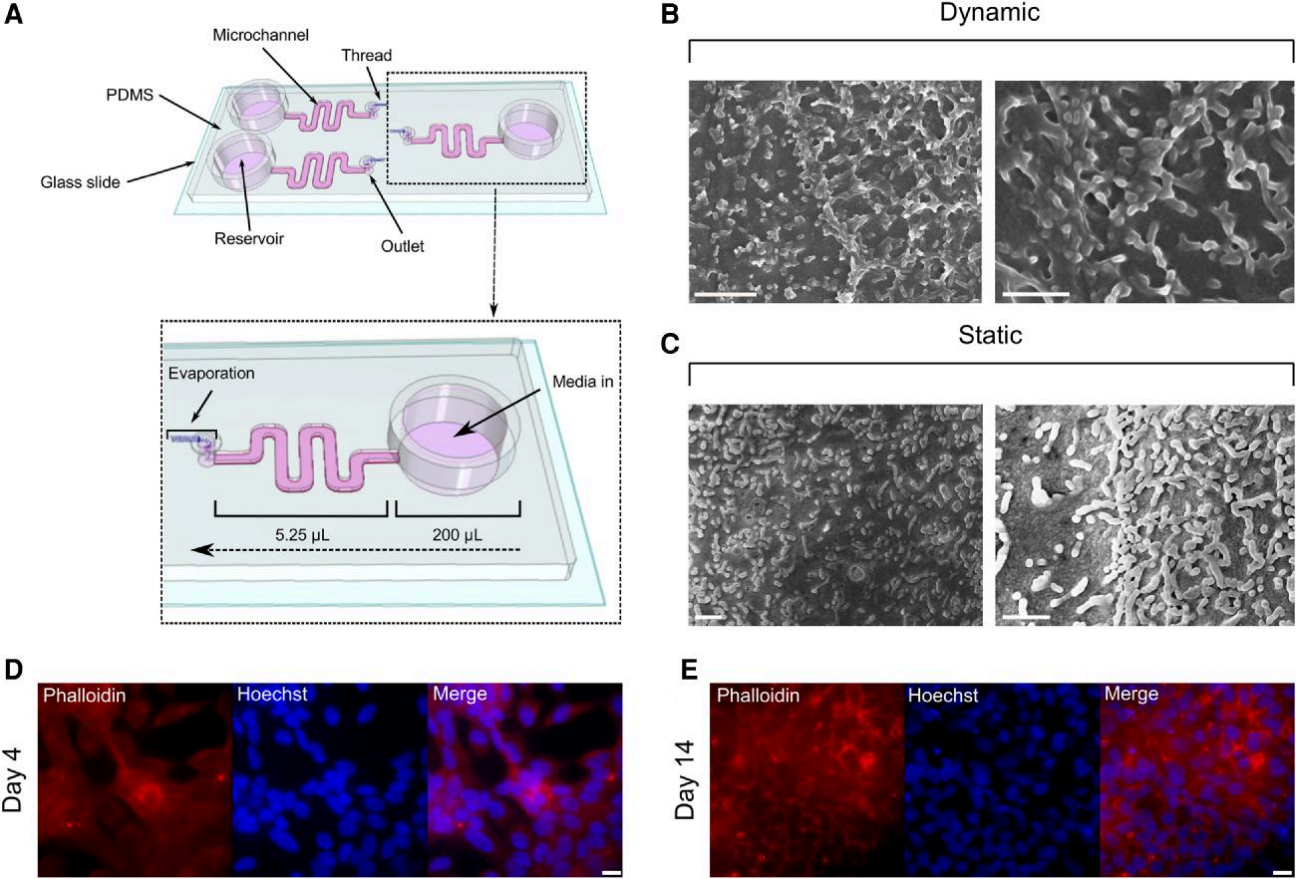
HCT-8 cells cultured under fluid shear stress within the microfluidic device enable enhanced formation of microvilli-like structures. A, Schematic image of the microfluidic device indicating the location of each microchannel, reservoir, outlet, and thread placement (diagram not drawn to scale). B, Scanning electron micrographs of the HCT-8 cell monolayer within the microfluidic device. HCT-8 cells were grown in Matrigel-coated microchannels under shear stress at 0.02 dyn cm^−2^ until 90% confluent (up to 5 days). The cell monolayers were then fixed with glutaraldehyde, subjected to graded ethanol and chemical dehydration, and coated with 5 nm of platinum. Data were acquired using a field emission scanning electron microscope. Representative images are shown. Scale bars = 2 µm (left), 1 µm (right). C, Scanning electron micrographs of the HCT-8 cell monolayer grown on borosilicate glass coverslips. Cells were grown until 90% confluent (up to 3 days) and prepared and imaged as described above. Scale bars = 1 µm. D, Representative fluorescence images of HCT-8 monolayers stained for F-actin (TRITC-phalloidin) and cell nuclei (Hoechst 33342) at 4 days post-plating. Images were captured using an IX83 inverted fluorescence microscope (Olympus) using the 100× objective lens. Scale bar = 20 µm. E, Representative fluorescence images of HCT-8 monolayers stained for F-actin (TRITC-phalloidin) and cell nuclei (Hoechst 33342) at 14 days post-plating. Images were acquired as described above. Scale bar = 20 µm. Abbreviation: HCT-8, human ileocecal colorectal adenocarcinoma.

This study utilized a microfluidic device with a pumpless and tubeless design using a protocol described previously [33]. Each device consisted of a glass coverslip bonded to a PDMS layer containing 3 microchannels (L: 36 mm; W: 1 mm; H: 155 µm) (Figure 1A). For devices undergoing preparation for scanning electron microscopy, a thicker microscope glass slide was bonded to the PDMS layer instead of cover glass. The total volume of each microchannel was 5.25 µL, and the reservoir volume was 200 µL. Before seeding, each microchannel was flushed with 70% (v/v) ethanol, then phosphate-buffered saline (PBS) pH 7.4 (Gibco) 3 times each. A 1% (v/v) Matrigel solution (Corning) diluted with RPMI-1640 (Sigma) was used to coat each microchannel by dispensing the solution through the outlet at 4°C, then incubating for 60 minutes at 37°C and 5% (v/v) CO_2_ in a humidified incubator.

### HCT-8 Cell Culture Inside the Microfluidic Device

The HCT-8 cell line (ATCC CCL-244) was routinely cultured in RPMI-1640 supplemented with 10% (v/v) fetal calf serum (FCS, Bovogen), 1% (v/v) penicillin-streptomycin (Sigma), 2 mM of L-glutamine (Sigma), and 15 mM of HEPES (Sigma), pH 7.2. The cell line was confirmed negative for *Mycoplasma* before experiments using a protocol described previously [34]. Cells were harvested from culture flasks using 0.25% (w/v) trypsin-EDTA (Sigma) and seeded into Matrigel-coated microchannels via the outlet at a concentration of 2 × 10^5^ cells per microchannel. The microfluidic devices were incubated overnight at 37°C with 5% (v/v) CO_2_ without the thread to facilitate cell adherence. The reservoir of each microchannel was then filled with RPMI-1640 supplemented as described above. To initiate fluid flow, a presterilized, hand-cut 10-mm spunlace thread (70% rayon, 30% polyester, Ebos Healthcare Australia) was inserted into the outlet of each microchannel using sterile, fine-tipped forceps. Cell monolayers were then grown to ∼90% confluence under shear stress (0.02 dyn cm^−2^) for downstream experiments. For static comparisons, the HCT-8 cell line was seeded at a concentration of 5 × 10^5^ cells per well into 24-well plates and grown to ∼90% confluence. The confluence and integrity of HCT-8 cell monolayers grown under both dynamic and static conditions were assessed qualitatively using phase contrast microscopy.

The morphology of the HCT-8 monolayer cultured within the microfluidic device was assessed at day 4 and day 14 post-seeding by staining the actin filaments and nuclei. Briefly, the monolayers were fixed at both time points with 1% (v/v) formalin for 30 minutes, followed by rinsing with 70% (v/v) ethanol. Cells were then washed with PBS, permeabilized with 0.05% IGEPAL (Sigma) for 20 minutes, washed again with PBS, and then treated for 30 minutes using a blocking buffer comprising 4% (v/v) normal goat serum (NGS; Thermo Fisher Scientific), 5% (v/v) FCS, and 0.1% (w/v) bovine serum albumin (Sigma) in PBS. The monolayers were then stained with 1 µg/mL of TRITC-Phalloidin (Sigma) and 5 µg/mL of Hoechst 33342 (Thermo Fisher Scientific) in 2% (v/v) NGS to stain actin filaments and nuclei, respectively. All incubations were performed at room temperature. Representative images from each time point were captured using an Olympus IX83 inverted fluorescence microscope.

### Excystation Pretreatment of Oocysts, Sporozoite Purification, and Infection of HCT-8 Cells

The *C. parvum* (Iowa-IIaA17G2R1) oocysts were obtained from BioPoint Pty Ltd. (Sydney, Australia), and the subtype was confirmed using a nested polymerase chain reaction (PCR) targeting the 60-kilodalton glycoprotein (*gp60*) locus described previously [35, 36], with minor modifications to the reagent and primer concentrations as reported elsewhere [37]. All experiments were undertaken using *C. parvum* oocysts within 12 weeks of receipt. The *C. parvum* oocysts underwent excystation pre-treatment as described previously [38]. Oocysts were resuspended in 20 µL of infection medium per microchannel, which comprised RPMI-1640 supplemented with 2 mM of L-glutamine, 5.6 mM of glucose, 0.02% (w/v) bovine bile, 15 mM of HEPES, 0.6 µM of folic acid, 7.3 µM of 4-aminobenzoic acid, 2.1 µM of calcium pantothenate, 50 µM of L-ascorbic acid, 2.5 µg/mL of amphotericin B, 1% (v/v) penicillin-streptomycin (all from Sigma), and 1% (v/v) FCS (Bovogen), pH 7.2. For experiments designed to detect new oocyst production within the microfluidic device, purified sporozoites were used for infections. The excystation-pretreated oocysts were resuspended in 1 mL of infection medium and then incubated at 37°C for 30 minutes, followed by filtration with a 2-µm syringe filter (Whatman). The syringe filter was rinsed with an additional 2 mL of infection medium, and then all filtrate-containing purified sporozoites were centrifuged at room temperature at 3200 *× g* for 20 minutes and resuspended in a precalculated volume of infection medium.

For infection, the spunlace thread was removed from each outlet, and then excystation-pretreated oocysts were applied to HCT-8 monolayers via the outlet at concentrations of 1 × 10^4^ oocysts or 4 × 10^5^ sporozoites per microchannel in a volume of 20 µL of infection medium. The devices were then incubated at 37°C and 5% (v/v) CO_2_ for 4 hours without the thread, after which fluid flow was re-initiated by inserting a fresh thread into the outlet of each microchannel. Infection medium from each microchannel and reservoir was replaced at 24-hour intervals. For static comparison experiments, excystation-pretreated *C. parvum* oocysts were applied to HCT-8 monolayers and cultured in 24-well plates at a concentration of 1 × 10^4^ oocysts per well.

### Parasite Enumeration Using Immunofluorescence Assays (Sporo-Glo Ab) and Quantitative PCR

For experiments designed to quantify parasite multiplication over time, a total of 16 microchannels were seeded with HCT-8 cells, and 15 were infected with excystation-pretreated *C. parvum* oocysts as described above. The uninfected microchannel seeded with HCT-8 cells functioned as a negative control for immunofluorescence assays and as a no template control (NTC) for quantitative PCR assays. Three microchannels were prepared for immunofluorescence assays at 48-hour intervals throughout the experiment. At each time point, the thread was removed from each outlet, submerged in 20 µL of infection medium, and stored at 4°C. The infection medium inside each microchannel and reservoir was then collected via the outlet and stored in 1.5-mL tubes at 4°C. For static comparisons, 3 wells per plate were allocated to each time point. The microfluidic devices and conventional cell culture plates were prepared for immunostaining as described previously [39]. A fluorescein-labeled anti-*Cryptosporidium* polyclonal antibody (1X Sporo-Glo, Waterborne Inc.) was administered to each microchannel directly from the outlet, or to each well, at a volume of 100 µL and incubated in the dark at room temperature for 60 minutes. After immunostaining, the remaining Sporo-Glo was aspirated, and then the cell monolayers were rinsed 4 times using PBS and imaged using a Nikon Eclipse TS100 inverted fluorescence microscope.

Following image acquisition, cell monolayers were harvested from each microchannel and each well using 0.25% (w/v) trypsin-EDTA (Sigma). The trypsin-EDTA was inactivated with an equal volume of infection medium and transferred to 1.5-mL tubes. All samples collected throughout experiments from harvested monolayers, threads, and residual infection medium from each time point were centrifuged at 20 000 *× g* for 10 minutes, and the supernatant was removed from each tube, leaving ∼20 µL remaining. All samples were freeze-thawed 5 times using alternating 60-second incubation periods in liquid nitrogen and 120-second incubation periods on a heating block set to 100°C. On the final thaw, each sample was boiled at 100°C for 5 minutes, and then the lysate was stored at −20°C until ready for use.

A quantitative PCR assay targeting a *Cryptosporidium*-specific gene encoding a C-type lectin protein was used to measure parasite load over time. The primer set (forward primer: EVF1 5′-GAA CTG TAC AGA TGC TTG GGA GAA T; and reverse primer: EVR1 5′-CCTT CGT TAG TTG AAT CCT CTT TCC A) amplified a 150-bp product and was used with a minor groove binding (MGB) probe (5′JOE – CTT GGA GCT CGT ATC AG – MGBNFQ) that was specific to *C. parvum* [40]. DNA was amplified using a CFX96 Touch Real-Time PCR Detection System (Bio-Rad) in a 10-µL reaction containing 1× GoTaq Probe qPCR Master Mix (Promega), 800 nm of EVF1 forward primer, 800 nm of EVR1 reverse primer, 250 nm of *C. parvum* MGB probe, and 2 µL of lysate diluted to 1:100. The reactions were carried out under the following cycling conditions: 95°C for 2 minutes, then 45 cycles of 95°C for 30 seconds and 60°C for 60 seconds. A standard curve was produced using a serially diluted gBlocks dsDNA Gene Fragment (Integrated DNA Technologies) that was synthesized to be homologous to the region of interest, with the AT-rich regions modified for increased GC content. Quantitative PCR data were acquired and analyzed using CFX Manager (Bio-Rad) and converted to oocyst equivalents on the basis that the gene is single copy [41, 42] and there are 4 haploid sporozoites per oocyst.

### Preparation of Microfluidic Devices for Scanning Electron Microscopy

A combination of uninfected HCT-8 monolayers and *C. parvum*–infected monolayers was prepared for imaging using scanning electron microscopy. The microchannels were coated with 1% (v/v) Matrigel, seeded with HCT-8 cells, and grown to confluence as described above. For static comparisons of the HCT-8 monolayer, cells were grown on 10-mm borosilicate glass coverslips instead. Each microchannel was then infected with 1.0 × 10^5^ excystation-pretreated *C. parvum* oocysts. The microchannels were rinsed 3 times with PBS and fixed overnight in 2.5% (v/v) glutaraldehyde diluted in 100 mM of HEPES buffer (both from Sigma), pH 7.4. For *Cryptosporidium*-infected microchannels, fixation occurred at 48 hours post-infection, whereas uninfected HCT-8 monolayers underwent fixation once the monolayer reached 90% confluence. The glutaraldehyde was rinsed off using 100 mM of HEPES buffer, keeping the entire device fully submerged in aqueous solution. The PDMS layer of the microfluidic device was removed from the microscope slide using a scalpel blade, and then each sample was subject to graded ethanol and hexamethyldisilazane dehydration. Samples were then sputter-coated with 5 nm of platinum. Morphological data were acquired using a Zeiss Supra 55VP field emission scanning electron microscope using the in-lens secondary electron detector and an accelerating voltage of 5 kV. The *C. parvum* life cycle stages were identified using morphological criteria described previously [43].

### Detection of New Oocysts Produced In Vitro

For experiments designed to detect new oocysts produced in vitro, 6 microchannels were seeded with HCT-8 cells as described above. Three microchannels were infected with purified *C. parvum* sporozoites, and the remaining microchannels were designated the negative control for immunofluorescence assays. All infection medium inside each microchannel and reservoir was collected in 1.5-mL tubes at 24-hour intervals for 7 days in total. All samples were centrifuged at 8000 *× g* for 3 minutes and washed twice with PBS. Samples were then prepared for immunostaining with EasyStain (BioPoint Pty Ltd.) using the supplier's instructions and imaged using a Nikon Eclipse 80i Fluorescence Microscope. To determine whether the in vitro–produced oocysts were infectious, the infection medium from 1 microchannel at 3 days post-infection underwent excystation pretreatment before application to a fresh HCT-8 monolayer. After 48 hours, the HCT-8 monolayer was prepared for immunostaining using Sporo-Glo as described above and imaged using a Nikon Eclipse TS100 inverted fluorescence microscope.

## RESULTS

**Figure 2.**
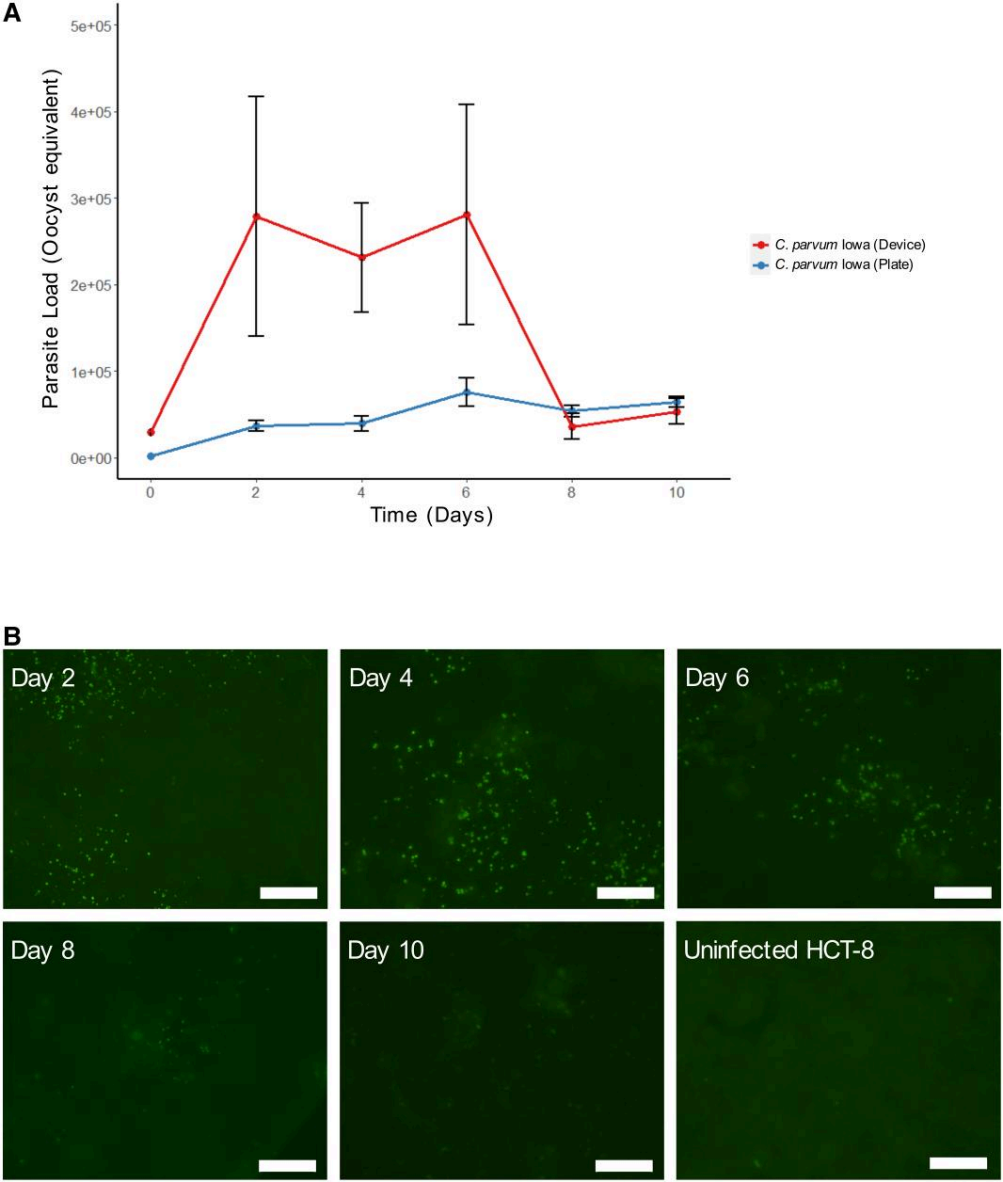
The fluid shear stress conditions of the microfluidic device enable extended *C. parvum* development. A, Quantitative PCR of *C. parvum* infection of the HCT-8 monolayer cultured within the microfluidic device vs conventional cell culture plate. HCT-8 cells were infected with excystation-pretreated *C. parvum* oocysts, and the infection medium, thread, and monolayer were sampled at 48-hour intervals. The overall parasite load was calculated from a standard curve and normalized for the total volume of the original, undiluted lysate. Results represent means ± SDs from n = 3 independent experiments performed in triplicate under fluid shear stress in the microfluidic device and n = 1 independent experiment performed in triplicate under static conditions on conventional cell culture plates. B, HCT-8 cells cultured under fluid shear stress infected with excystation-pretreated *C. parvum* Iowa oocysts, then fixed and stained with Sporo-Glo (fluorescein-labeled polyclonal anti-*Cryptosporidium* antibody) at each time point from 2 to 10 days post-infection (scale bars = 100 µm). Abbreviations: HCT-8, human ileocecal colorectal adenocarcinoma; PCR, polymerase chain reaction.

This study reports the in vitro development of *C. parvum* in HCT-8 cell monolayers grown within the microfluidic device for up to 10 days. HCT-8 cells cultured at 0.02 dyn cm^−2^ exhibited well-developed microvilli-like structures compared with the same cells cultured within conventional cell culture plates (Figure 1B, C). The monolayer remained intact over this time frame, as indicated by phalloidin staining of the actin cytoskeleton (Figure 1D). HCT-8 cells cultured within the microfluidic device were challenged with excystation-pretreated *C. parvum* oocysts, and sampling was undertaken at 48-hour intervals from the cell culture medium within the microchannel, the absorbent thread initiating fluid flow, and the cell monolayer harvested from each microchannel. No visual cytopathic effect was observed by phase contrast microscopy throughout the infection protocol (data not shown). Quantitative PCR values for these samples were pooled together as the total parasite load (referred to as oocyst equivalents) at each time point (Figure 2A).

The quantitative PCR data were supported by immunofluorescence assays using the Sporo-Glo polyclonal antibody, which indicated that foci of infection were present in the HCT-8 monolayer at each time point up to 10 days post-infection (Figure 2B). No immunostaining was detected in the uninfected HCT-8 monolayers. The number of foci visible in the monolayers declined rapidly from 8 to 10 days post-infection, which coincided with what was observed from the quantitative PCR data.

**Figure 3.**
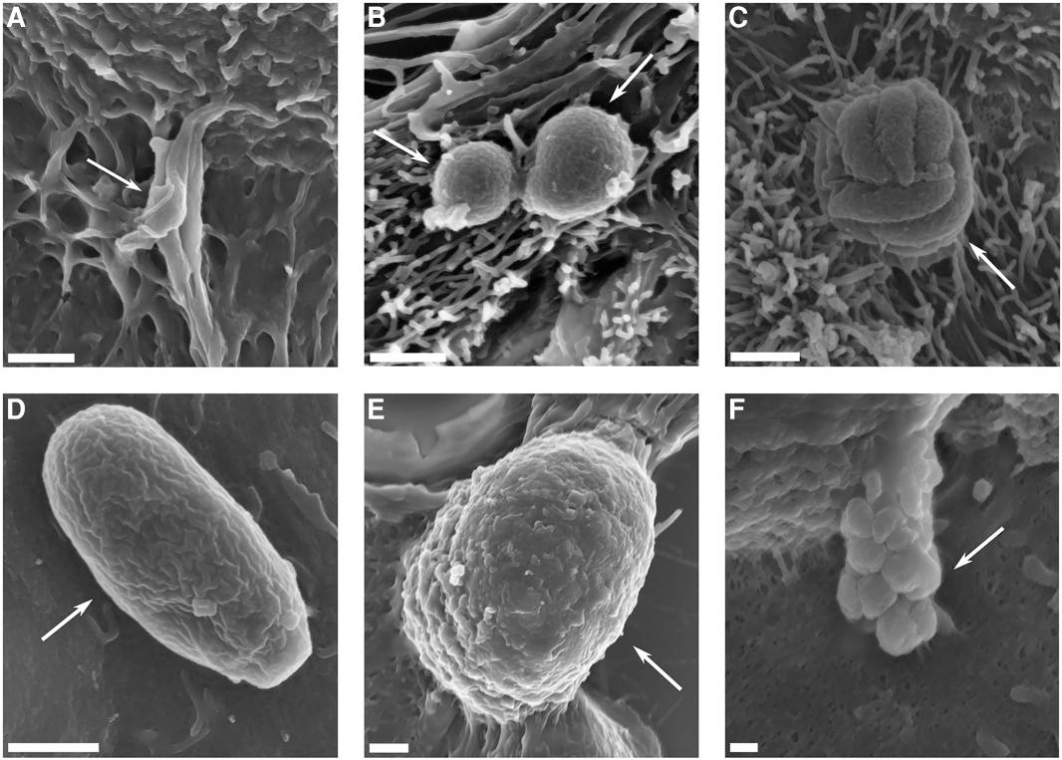
Scanning electron micrographs of *C. parvum* infecting the HCT-8 cell line grown under fluid shear stress conditions at 2 days post-infection. A, Sporozoite invading the host cell monolayer. B, Trophozoites adhered to the host cell monolayer appearing as spherical structures on the cell surface. C, Meront adhered to the host cell. D, Merozoite attached lengthwise to the apical surface of the host cell. E, Macrogamont attached to the host cell. F, Microgamont hanging from a stalk with multiple microgametes visible. Scale bars: A–E = 1 µm; F = 200 nm. Abbreviation: HCT-8, human ileocecal colorectal adenocarcinoma.

Scanning electron micrographs of the *Cryptosporidium*-infected microfluidic devices at 2 days post-infection demonstrated the presence of all common life cycle stages, including both asexual and sexual phases of development. The scanning electron micrographs indicated the presence of sporozoites, trophozoites, meronts, and merozoites attached to the host cell surface. Microgamonts and macrogamonts were visible at this time point (Figure 3). The invasive life cycle stages (sporozoites, merozoites) were observed rarely. Trophozoites and meronts were regularly observed. Of the sexual stages, macrogamonts were more frequently observed than microgamonts.

**Figure 4.**
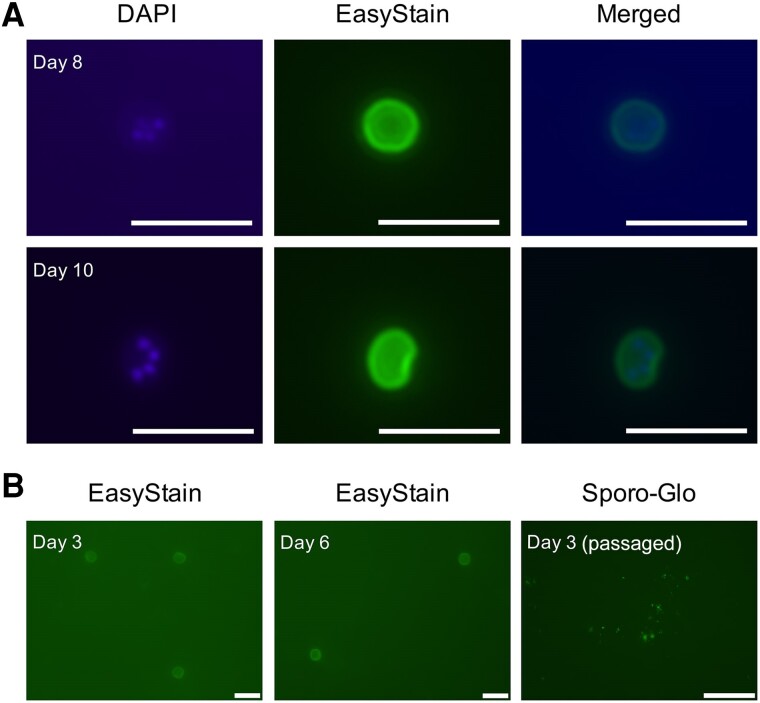
The microfluidic device enables formation of *C. parvum* oocysts that are infectious to fresh HCT-8 monolayers. A, Contents of 1 microfluidic device from 8 days and 10 days post-infection stained with DAPI and EasyStain and merged image for both time points. B, Left 2 images are the contents of 1 microfluidic device from 3 days and 6 days post-infection and stained with EasyStain for both time points. The image on the right is an HCT-8 monolayer with the contents of 1 microchannel at 3 days post-infection, applied to the fresh monolayer and cultured in a conventional cell culture plate for 48 hours before immunostaining with Sporo-Glo. Scale bars: A = 10 µm; B = 10 µm (left); 100 µm (right). Abbreviations: DAPI, 4′,6-diamidino-2-phenylindole; HCT-8, human ileocecal colorectal adenocarcinoma.

A commercially obtained antibody that binds to *Cryptosporidium* oocyst walls (EasyStain) was used with 4′,6-diamidino-2-phenylindole (DAPI) to identify the presence of newly formed *Cryptosporidium* oocysts within the microfluidic device. The EasyStain immunofluorescence assay indicated that oocysts were present in low numbers in the cell culture medium of the *C. parvum*–infected microchannels from 3 to 10 days post-infection (Figure 4), and oocyst shells were present in the cell culture medium at 1 day post-infection. The oocysts that were detected in the cell culture medium often did not stain with DAPI (Figure 4). Oocyst shells were observed, but rarely and in very low numbers when sampled immediately post-filtration and at 2 days post-infection. When the cell culture medium from a ­*C. parvum*–infected microchannel at 3 days post-infection was applied to a fresh HCT-8 monolayer, foci of infection were observed at 48 hours post-infection of the passaged culture, indicating that the oocysts produced in vitro within the microfluidic HCT-8 cell cultures were infectious to a fresh HCT-8 cell monolayer (Figure 4).

## DISCUSSION

This study demonstrated the in vitro development of *C. parvum* using the HCT-8 cell line for up to 10 days using a pumpless and tubeless microfluidic device. Our data indicated an increase in parasite load over time, with the infection peaking at 2 days post-infection, maintained until 6 days post-infection, and then declining rapidly from day 8 to day 10. The quantitative PCR data were supported by immunofluorescence data indicating robust infection up to 6 days post-infection, followed by low numbers of foci of infection at 8 days post-infection, which declined further by 10 days post-infection. Scanning electron micrographs demonstrated a large amount of heterogeneity of both asexual and sexual developmental stages after 2 days of infection under fluid shear stress conditions.

The seminal work of English et al. (2022) provided insights into the infection kinetics of *C. parvum*, where it was demonstrated that the parasite must undergo 3 cycles of asexual replication before sexual development will occur [44]. Within the conventional cell culture plate, fertilization is very limited, which results in cessation of *C. parvum* infection 3 days post-infection [20, 45]. The findings of the present work suggest that 1 cycle of successful sexual replication occurred within the microfluidic device, and excystation of oocysts produced in vitro occurred within the microchannel. The data suggest subsequent cycles of asexual development occurring after 3 days post-infection. The loss of reliability of the culturing system after 6 days of infection may coincide with a possible second round of sexual development resulting in a diminished number of oocysts produced in vitro to sustain infection within the microfluidic device and may also reflect the aging of the HCT-8 host cells. While the present study provided evidence of the production of new and viable oocysts in vitro at 3 days post-infection, we present insufficient evidence to indicate that the fertilization block observed in conventional HCT-8 cell culture is completely circumvented with the microfluidic gut-on-chip, given that the new oocysts were present in relatively low numbers. The presence of oocysts that would not stain with DAPI but were still infective to new cultures was unsurprising due to an unclear relationship between oocyst viability and permeability to vital dyes [46, 47]. In the in vivo environment, gut epithelial cells are exposed to a wide variety of mechanical stimuli that result in the application of shear stress, including peristaltic muscle contraction, intraluminal fluid flow, and rhythmic extension and contraction of the intestinal villi [48]. The application of fluid shear stress on gut epithelial cell lines cultured in vitro within microfluidic devices has been demonstrated to alter their phenotype, translating to a more physiologically relevant representation of the in vivo structure and function. Such phenotypical changes elicited by fluid shear stress in gut epithelial cells include increased cell height, improved density of F-actin networks, enhanced microvilli and tight junction formation, and elevated mucin production [29–31, 49–51]. Based the above information, we interpret the extended duration of *C. parvum* cultures using the microfluidic gut-on-chip to be primarily the result of the difference in phenotype of the HCT-8 cell line. Our scanning electron microscopy data demonstrated that the HCT-8 cell line exhibits improved microvilli when cultured in the microfluidic gut-on-chip compared with borosilicate glass slides; however, this observation may be partially attributed to the enhanced longevity of the HCT-8 cell cultures within the microfluidic gut-on-chip, given the positive relationship between cell culture age and differentiation observed in the HCT-8 cell line [52]. *Cryptosporidium* infection in humans is typically restricted to the apical brush border of the intestinal epithelium [53], further supporting the importance of enhanced host apical surface structure in a *Cryptosporidium* co-culture system. However, the possibility that the presence of fluid shear stress over the duration of the experiment played a role in supporting extended *C. parvum* development cannot be excluded based on the present study and should be further investigated in the future.

The majority of gut-on-chip microfluidic devices require integration of an external peristaltic pump and tubing to control perfusion of cell culture medium through the microchannels [54, 55]. In practice, these are prone to leakages [56], and therefore suboptimal for culturing *Cryptosporidium* due to the elevated biological hazard this creates. The pumpless and tubeless microfluidic device utilized in the present study minimizes this risk and can be easily integrated into existing workflows that do not routinely use microfluidics due to its simplified design. These devices can be easily prepared at a low upfront cost within basic microfabrication facilities (<AUD$1 per microfluidic device). Future studies should focus on understanding the upper limit of microfluidic gut-on-chips in terms of oocyst production and if these oocysts are capable of progressing through the life cycle to generate new oocysts when infected into microfluidic HCT-8 cultures. Increasing the complexity of the microfluidic culture system described in the present study by establishing apical and basal compartments [30] and introducing cyclic stretch of the partitioning membrane [31] are also potential avenues for future experimentation that may further enhance the longevity of the present culture system in supporting *C. parvum* development and may improve oocyst production. Given that many labs that study the in vitro development of *Cryptosporidium* already have robust HCT-8 plate-based culturing systems, this microfluidic device could integrate seamlessly into existing workflows due to its simplicity and extend the boundaries of what can currently be achieved with the conventional cell culture plate system.

Currently, the costs associated with routine *Cryptosporidium* infectivity testing using the HCT-8 cell line grown in standard cell culture plate wells limit their widespread usage within the water industry, at an estimated cost per sample of ∼AUD$400 for the *Cryptosporidium* culturing component alone. Forgoing *Cryptosporidium* infectivity testing due to cost limitations can lead to failures in accurately quantifying the risk of *Cryptosporidium* oocyst detections in source water and challenges in meeting health-based targets for water quality. Using microfluidic gut-on-chips instead of conventional cell culture plates for *Cryptosporidium* infectivity testing could contribute to minimizing the reagent costs due to the small volume of the microchannel (5.25 µL) and reservoir (200 µL) and would result in a total cost reduction of ∼17%. For laboratories wanting to apply this technology for routine *Cryptosporidium* infectivity studies, it could provide a more affordable system to determine the proportion of infectious oocysts recovered from drinking water and wastewater supplies using either the focus detection method with Sporo-Glo [39] or combination with quantitative PCR [40]. In the future, the microfluidic device described in the present study may form part of an integrated assay to quantitate *Cryptosporidium* oocyst viability and identify species and subtype to better inform water quality risk assessments.

## CONCLUSIONS

We have shown that an easy-to-use, inexpensive microfluidic device supports the growth of all commonly observed life cycle stages of *C. parvum* within HCT-8 cells for up to 10 days. We anticipate that this approach will make studies on *Cryptosporidium* infectivity, life cycle, host–parasite interactions, and high-throughput drug assays more accessible to both researchers and industry and contribute to the advancement of our understanding of the biology of *Cryptosporidium*.
